# Immunogenicity of twenty peptides representing epitopes of the hepatitis B core and surface antigens by IFN-γ response in chronic and resolved HBV

**DOI:** 10.1186/s12865-015-0127-7

**Published:** 2015-11-02

**Authors:** Nanna-Sophie Brinck-Jensen, Thomas Vorup-Jensen, Peter Derek Christian Leutscher, Christian Erikstrup, Eskild Petersen

**Affiliations:** Department of Infectious Diseases, Aarhus University Hospital, Palle Juul-Jensens Boulevard 99, 8200 Aarhus N, Skejby Denmark; Department of Biomedicine, Aarhus University, Wilhelm Meyers Allé 4, 8000 Aarhus C, Denmark; Department of Clinical Immunology, Aarhus University Hospital, Palle Juul-Jensens Boulevard 99, 8200 Aarhus N, Skejby Denmark

**Keywords:** Hepatitis B virus, T cell immunity, HLA genotypes, IFN-γ, ELISPOT, Epitope prediction

## Abstract

**Background:**

Patients with chronic hepatitis B virus infection (CHB) usually mount a modest T cell response against HBV epitopes. In order to determine immunogenic epitopes of HBV recognized by HBV-specific T cells, previous studies focused on previously confirmed HBV epitopes and assessed the T cell response by the number of HBV-specific T cells by IFN-γ ELISPOT.

**Methods:**

We studied T cell functionality by combined in silico methods predicting HBV-specific epitopes and experimental investigations on the recognition of these epitopes. 30 chronic CHB patients and 10 patients with resolved HBV (RHB) were included in the study. We identified epitopes from the literature and by in silico analysis. These were evaluated for immunogenicity by use of synthetic peptides representing the epitopes through exposure to PBMCs from patients with CHB or RHB by IFN-γ ELISPOT. The number of IFN-γ producing cells (SFC), mean spot size (MSS) and stimulation index (SI) were recorded.

**Results:**

The frequency of HBV-specific T cells producing IFN-γ after stimulation with HBV epitopes was similar in CHB and RHB patients. CHB patients had a higher MSS SI than RHB patients. Patients not carrying the HLA-A2 genotype had higher SFC SI and MSS SI. Patients with HLA-A11 had higher MSS SI compared to non- HLA-A11 allele patients. HBeAg-positive patients had a lower MSS SI, and none of the HBeAg positive patients had the HLA-A11 genotype. We found 3 immunogenic epitopes not described previously.

**Conclusion:**

IFN-γ ELISPOT-determined MSS is an efficient marker for T cell recognition of epitopes. This experimental measure showed the in silico analysis for epitope prediction to be a valuable tool in future studies on HLA genotypes and HBV epitopes. This way our study now points to previously unappreciated consequences of carrying the HLA-A11 allele in terms of stronger immunity to HBV.

## Background

Hepatitis B virus (HBV) is the cause of a spectrum of acute diseases including fatal hepatocellular necrosis. More than 350 million people have chronic HBV infection, which causes approximately one million deaths per year from the associated morbidities, liver cirrhosis and cancer [1]. Several lines of evidence suggest that HBV infection induces suppression of the immune response to the viral components as a result of both viral tropism and antigen release.

In patients with chronic HBV infection HBV-specific T cells egress into the liver but present only a modest anti viral response [2, 3]. By contrast, patients with an acute, self-limited HBV infection usually mount a vigorous polyclonal CTL response targeting multiple HBV epitopes [4] and with sufficient longevity to be detectable several years after infection [5, 6]. Here we show that this is a conclusion not always cogent in the case of patients with a resolved HBV more than two years ago.

As part of the studies to understand the immune response to HBV infection, an important part of the efforts has been focused on identifying highly immunogenic CTL epitopes. One approach involves in silico predictions on the dissociation constant (*K*_D_) for the binding of antigen-derived peptides to the Human Leukocyte Antigen (HLA) molecules, typically accompanied by experimental testing of CTL response to these peptides or experimentally tested ability of HLA molecules to bind these peptides. A number of studies reported that binding between HLA molecules and antigenic peptides characterized by *K*_D_ <500 nM appears to enable CTL responses [7]. However, other studies suggest that the effects of antigenic epitopes not always correlate with the affinity of the epitope-containing peptide to HLA [8, 9]. Previous studies evaluated the immune response to HBV infection by testing previously confirmed HBV epitopes and assessed the T cell response by determining the frequency of reactive T cells, typically by IFN-γ enzyme-linked immunosorbent spot (ELISPOT) analysis. From such studies it was suggested that the frequencies of antigen-specific CTLs may not be the major determinant of immune-mediated protection in chronic hepatitis B, nor should immunotherapeutic approaches only aim at raising the frequency of HBV-specific T cells. Indeed, T cell functionality, such as ability to produce cytokines, may also be important parameters [10]. However, the correlation between HLA affinity for epitope-containing peptides and T cell cytokine production as estimated by ELISPOT has not been comprehensively studied in the context of chronic HBV infection.

### Objectives

Here, we combine in silico methods for predicting HBV-specific CTL epitopes and compare with the T cell response in vitro. By comparing cells from patients with chronic HBV infection (CHB) and patients with resolved HBV infection (RHB), we show that the calculations were efficient in predicting immunogenic epitopes recognized by T cells from CHB patients. Indeed, T cell functionality calculated from the amount of IFN-γ produced was increased in CHB patients with HLA-A11 genotype.

## Methods

### Patients

The study population consisted of patients with CHB, i.e., positive HBsAg status for more than six months, followed in Aarhus University Hospital, and former patients with RHB previously seen in Aarhus University Hospital. The diagnosis of acute HBV was based on clinical and biochemical evidence of acute liver injury according to standard diagnostic criteria of acute HBV infection, i.e. elevated liver enzymes, positive HBsAg and IgM-antibodies against HBcAg [11] and reviewed in [12]. Written consent was obtained from each participant, including consent to publish all personal information contained in Table 3. The study was approved by the The Central Denmark Region Committees on Health Research Ethics, ref. number M-40-12 and the National Data Protection Agency journal number 2012-41-0028.

### Virological analyses

HBsAg, HBeAg, anti-HBe, anti-HBc, anti-HBs, HIV Ag/Ab, and anti-HCV were determined by commercially available chemiluminescense assays on the Architect system (Architect, Abbott Laboratories, Abbott Park, Illinois, USA). HBV DNA levels were quantified by commercial hybridization assay.

### Peripheral blood mononuclear cells (PBMCs)

PBMCs were isolated from 50 mL of fresh whole blood in 6 × 8 mL cell preparation tubes prefilled with 1 mL 0.1 M sodium citrate and 3 g of polyester gel, 2.0 mL of FICOLL™ Hypaque™ solution (BD Vacutainer**®** CPT™). Pellet was resuspended in RPMI-1640 with 20 % (v/v) FCS and 10 % (v/v) DMSO and frozen in liquid nitrogen.

### HLA loci genotyping

High-resolution HLA class I typing was performed using Sequence Based Typing method with PCR sequencing templates performed on both strands. Sequencing DNA templates was produced by locus- and group-specific amplifications that include exon 2 and 3, which contain the antigen recognition sites. Class I sequencing primers where the common sequences for all loci in the intron/exon boundary regions and a total of 40 locus and group-specific primers were used to amplify the target sequences (HistoGenetics LLC, Ossining, NY, USA).

### Epitope selection

Published reports on acute and chronic HBV patient and response to various HBV epitopes was studied and compared to NetMHC version 3.2 predictions of 8-mer epitopes, using Artificial Neural Networks Approximation (www.cbs.dtu.dk/services/) [6, 8, 9, 13–19]. Computer predictions were made on class I genotypes HLA-A11, HLA-A24 and HLA-A2 since these genotypes covered the majority of our patientpopulation. The surface sequences vary greatly among different HBV genotypes and subtypes [20] and we observed single and double residue variations between the published sequences and sequences found in the database of the computer algorithm. For this reason we were not able to directly compare the findings of the published reports with the computer predictions. The computer predicted epitopes were designed by cross matching the different HLA subtypes and HBV genotypes of the study population. According to classifications made earlier [21], a dissociation constant *K*_D_ < 50 nM predicted strong binding, 50–500 nM weak binding, and *K*_D_ > 500 nM predicted essentially no binding. Analysis of protein parameters isoelectric point (pI) [22], length, instability index [23] and aliphatic index [24] were performed on each epitope (http://_web.expasy.org/protparam/_).

### Peptides mimetics of HBsAg-specific epitopes in ELISPOT assays

Twenty HBsAg and HBcAg epitopes were purchased from GL Biochem (Shanghai, China), with a purity > 95 % as estimated from mass spectometry. We used HLA class I-restricted T cell epitopes from human cytomegalovirus, Epstein Barr virus and influenza virus (CEF) for positive control (CTL-Europe GmbH, Germany).

Enzyme-linked immunospot (ELISPOT) assays were performed using the 20 different peptides seeded in separate wells. Briefly, 96-well plates (Multiscreen-IP; Millipore S.A.S., Molsheim, France) were coated overnight at 4 °C with 100 μL/well capture mouse anti-human IFN-γ monoclonal antibody (AH diagnostics, Aarhus, Denmark). Plates were washed twice with ELISPOT coating buffer and blocked with CTL serum-free media for 1–2 h at room temperature (CTL-Europe GmbH, Germany). PBMCs (3 × 10^5^/well) were thawed and suspended in CTL serum-free media then seeded in triplicate for each individual peptide. Plates were incubated for 48 h at 37 °C and washed with PBS and 0.05 % Tween-20. 100 μL/well biotinylated secondary mouse anti-human IFN-γ monoclonal antibody was added according to manufacturer recommendations. After 2 h incubation at room temperature, plates were washed four times with PBS and 0.05 % Tween-20, 100 μl of Avidin-HRP solution was added to the wells, and the plates were incubated for further 45 min at room temperature. Plates were washed 3 times with PBS and 0.05 % Tween-20, and 2 times with PBS alone, and 100 μl of AEC substrate solution (3-amino-9-ethyl carbazole) was added. After 10–15 min, the colorimetric reaction was stopped by washing three times with distilled water. Plates were air dried, and spots were counted and analyzed using an automated ELISPOT reader (CTL-Immunospot S6 Analyzer, CTL GmbH, Germany). The T cell response was assessed as spot forming cells (SFC) and mean spot size (MSS). To exclude subjective assessment, whether the background were related to the actually response, we decided to use the stimulation index (SI) for analysis, calculated by dividing the value of the stimulated sample with the value of the unstimulated control. Four uninfected individuals (with clean HBV serology) were included to exclude unspecific findings (data not shown). All patients responded vigorously to the positive CEF control.

### Statistics

Statistical analysis was performed using GraphPad Prism 6 (©2014 GraphPad Software, Inc) and STATA/IC 13.1 (©2014 StataCorp, LP). Non-parametric measures were analysed by Spearman’s correlation, Mann–Whitney *U* test, Kruskal-Wallis test with Dunn’s correction for multiple comparison while parametric data were assessed by Student’s *T*-test and one-way analysis of variance (ANOVA).

## Results

### HLA genotypes and epitopes

Ten experimentally confirmed (EC) HBsAg and HBcAg epitopes were chosen from the literature, and 10 8-mer HBsAg and HBcAg epitopes were chosen based on the computer predictions (CP) of HLA class I binding to the epitopes (Tables 1 and 2). Among CHB patients, 12/30 (40 %) were HLA-A*02:01, 1/30 (3 %) HLA-A*02:03, 1/30 (3 %) HLA-A*02:06 and 1/30 (3 %) HLA-A*02:17. Among RHB patients, 6/10 (60 %) were HLA-A*02:01. We included one surface epitope tested among patients with HLA-A*11 genotype and one tested among HLA-A*24 genotype patients. Among the patients, 5/30 (17 %) CHB patients and 2/10 (20 %) RHB patients had HLA-A*11 genotype, and 6/30 (20 %) CHB patients had HLA-A*24 genotype. No RHB patients were HLA-A*24 genotype. Characteristics of patients are shown in Table 3.

**Table 1:** HLA class I restricted epitopes (I)

Number	Residues	Sequence	HLA	Reference
HBsEC-1	20–28	FLLTRILTI	A*02:01, 02:05, 02:06	[13]
HBsEC-2	88–96	LLCLIFLLV	A2	[9, 14]
HBsEC-3	95–104	LVLLDYQGML	A2	[8]
HBsEC-4	172–180	WLSLLVPFV	A*02:01, 02:02,02:03,02:05, 02:06	[6, 8, 9, 14, 15]
HBsEC-5	207–216	SIVSPFIPLL	A2	[14]
HBsCP-6^b^	169–176	FLGPLLVL	A2^a^	
	180–187			
	179–188			
HBsCP-7^b^	72–79	LLGWSPQA	A2^a^	
	61–68			
	71–78			
HBsCP-8^b^	259–266	LLLCLIFL	A2^a^	
	248–255			
	258–265			
HBsCP-9^b^	333–370	FLWEWASA	A2^a^	
	332–339			
	322–329			
HBsCP-10^b^	129–136	PAGGSSSG	A2^a^	
	140–147			
	139–146			
HBcEC-11	18–27	FLPSDFFPSV	A*02:01, 02:05, 02:06	[6, 8, 9, 14–17]
HBcEC-12	88–96	YVNVNMGLK	A11	[18]
HBcEC-13	107–115	CLTFGRETV	A2	[8]
HBcEC-14	117–125	EYLVSFGVW	A24	[19]
HBcEC-15	139–148	ILSTLPETTV	A2	[8]
HBcCP-16	141–148	TLPETTVV	A2	
HBcCP-17	160–168	PSPRRRRS	A2	
HBcCP-18	64–71	LMTLATWV	A2	
HBcCP-19	29–36	LLDTASAL	A2	
HBcCP-20	107–114	LTFGRETV	A2	

Name and amino acid position of the 20 epitopes. HLA genotype indicates which genotypes were tested in previous studies (HBsEC1-5 and HBcEC11-15), or which genotypes we stratified for in the in silico analysis (HBsCP6-10 and HBcCP16-20)

*HBsEC* HBsAg experimentally confirmed, *HBsCP* HBsAg computer predicted, *HBcEC* HBcAg experimentally confirmed, *HBcCP* HBcAg computer predicted

^a^all subtypes

^b^differences of sequence position among HBV genotypes was observed for in silico prediction of HBsAg

**Table 2:** HLA class I restricted epitopes (II)

Number	Response AHB	Response CHB	*K*_D_	Reference
HBsEC-1	14/23	N/A	N/A	[13]
HBsEC-2	6/13	1/15	N/A	[9, 14]
HBsEC-3	N/A	16/17	N/A	[8]
HBsEC-4	42/56	5/33	N/A	[6, 8, 9, 14, 15]
HBsEC-5	2/4	0/12	N/A	[14]
HBsCP-6			<50 nM	
HBsCP-7			<50 nM	
HBsCP-8			<50 nM	
HBsCP-9			<50 nM	
HBsCP-10			>500 nM	
HBcEC-11	41/59	9/30	50–10,000 nM	[6, 8, 9, 14–17]
HBcEC-12	N/A	1/1	N/A	[18]
HBcEC-13	N/A	2/5	>50 nM	[8]
HBcEC-14	7/12	0/11	>500 nM	[19]
HBcEC-15	N/A	15/18	>50 nM	[8]
HBcCP-16			<50 nM	
HBcCP-17			>500 nM	
HBcCP-18			<50 nM	
HBcCP-19			<50 nM	
HBcCP-20			50–500 nM	

All *K*_D_ values are in silico predictions by NetMHC version 3.2 8mer predictions. HBsEC: HBsAg experimentally confirmed; HBsCP: HBsAg computer predicted; HBcEC: HBcAg experimentally confirmed; HBcCP: HBcAg computer predicted. N/A: Not available, AHB and CHB referres to number of responders in previous studies of acute hepatitis B (AHB) and chronic hepatitis B patients (CHB)

**Table 3:** Patient characteristics

ID	Age	Sex	Ethnicity	HLA class I	HBeAg
C1	57	F	Black African	A2A24 B44B58 C3C16	Neg
C2	46	F	Asian	A24A31 B35B51 C1C14	Neg
C3	43	M	Arab	A2A3 B44B47 C6C16	Neg
C4	35	M	Asian	A11A33 B44B55 C3C7	Neg
C5	52	M	Asian	A2A33B40B51 C14C15	Pos
C6	33	M	White European	A2 B14B18 C7C8	Neg
C7	49	F	Asian	A1A11 B35B52 C4C7	Neg
C8	39	F	White European	A3A74B15B35 C4	Neg
C9	29	F	White European	A1A32 B40B44 C3C16	Neg
C10	65	M	White European	A2A29 B7B44 C7C16	Pos
C11	30	F	White European	A2 B38B51 C12C15	Neg
C12	26	F	White European	A2A24 B18B38 C7C12	Neg
C13	43	F	Asian	A2A26 B56B59 C1C7	Neg
C14	46	M	White European	A1A2 B37B51 C6C15	Neg
C15	73	M	White European	A2A3 B7 C7	Neg
C16	53	M	Asian	A11A24 B13B18 C3C7	Neg
C17	29	F	White European	A2 B13 C6	Neg
C18	61	F	Asian	A11A33 B13B58 C3	Neg
C19	40	F	Asian	A11A33 B44B59 C1C14	Neg
C20	51	F	Black African	A29A30 B18B45 C6C7	Pos
C21	34	F	Asian	A24A26 B13B40 C3C6	Pos
C22	30	M	White European	A2A26 B38B51 C12C15	Neg
C23	62	F	White European	A2A68 B15B44 C3C7	Neg
C24	51	M	White European	A2A3 B7B18 C7	Neg
C25	61	M	White European	A2A26 B7B51 C1C12	Neg
C26	34	F	Asian	A24A26 B13B40 C3C6	Pos
C27	61	F	White European	N/A	Neg
C28	49	M	Asian	N/A	Neg
C29	37	M	Asian	N/A	Pos
C30	23	M	White European	N/A	Neg
R31	40	F	White European	A2 B15B44 C1C5	
R32	30	F	White European	A2A68 B35B44 C4C5	
R33	N/A	N/A	White European	A2A26 B38B40 C12C15	
R34	48	M	White European	A23A26 B35B44 C4	
R35	65	F	White European	A1A2 B7B8 C7	
R36	43	F	White European	A1A11 B8B15 C7	
R37	38	M	White European	A2A11 B15B55 C3	
R38	69	F	White European	A2 B7B8 C7	
R39	73	F	White European	N/A	
R40	25	F	White European	N/A	

Normal ALT levels were found in all patients except patient C18 and C19 where ALT levels were elevated to 59 and 66 IU/mL (normal range for females is 10–45 IU/mL). No patients were double positive or double negative for HBeAg/anti-HBeAg. Median HBV DNA in the HBeAg positive patients were 81,000 IU/mL (2 – 400.000 IU/mL) and 0.019 IU (0 – 5500 IU/mL) in the HBV negative patients, no statistics were made on HBV genotype since quantification was not possible in 12 of the 30 patients. N/A: not available; F: female; M: male; Pos: positive; Neg: negative

### IFN-γ ELISPOT

Age-related data of the CHB and RHB patients were tested for normality and equal SD and analysed by students *t*-test. No significant difference was found between the two groups (difference in year: −3.16 (CI: −14.1–7.8), *p* = 0.72). To test whether the response to HBV epitopes of the CHB patients depended on age, ethnicity and gender, Kruskal Wallis oneway analysis of variance were performed on each separate response parameter, SFC and MSS. No significant difference of response was found among the four ethnic groups Black African (*n* = 2), Arab (*n* = 1), Asian (*n* = 12) and White European (*n* = 15), *p* = 0.68 for MSS, *p* = 0.12 for SFC. Furthermore no significant difference were found among males and females, *p* = 0.55 for MSS, *p* = 0.13 for SFC. We have previously reported that age is an important determinant in humoral and T cell responses to immunization with hepatitis B surface antigen [25] so to test whether age were related to the response to HBV epitopes, we divided patients in three groups: 23–30 years (*n* = 6), 31–49 years (*n* = 13) and 50–73 (*n* = 11). No significant difference of response were found between the groups, *p* = 0.15 for MSS, *p* = 0.45 for SFC. Due to the limited number of patients in the RHB group, the statistical power was not strong enough to make subanalysis.

**Fig. 1:**
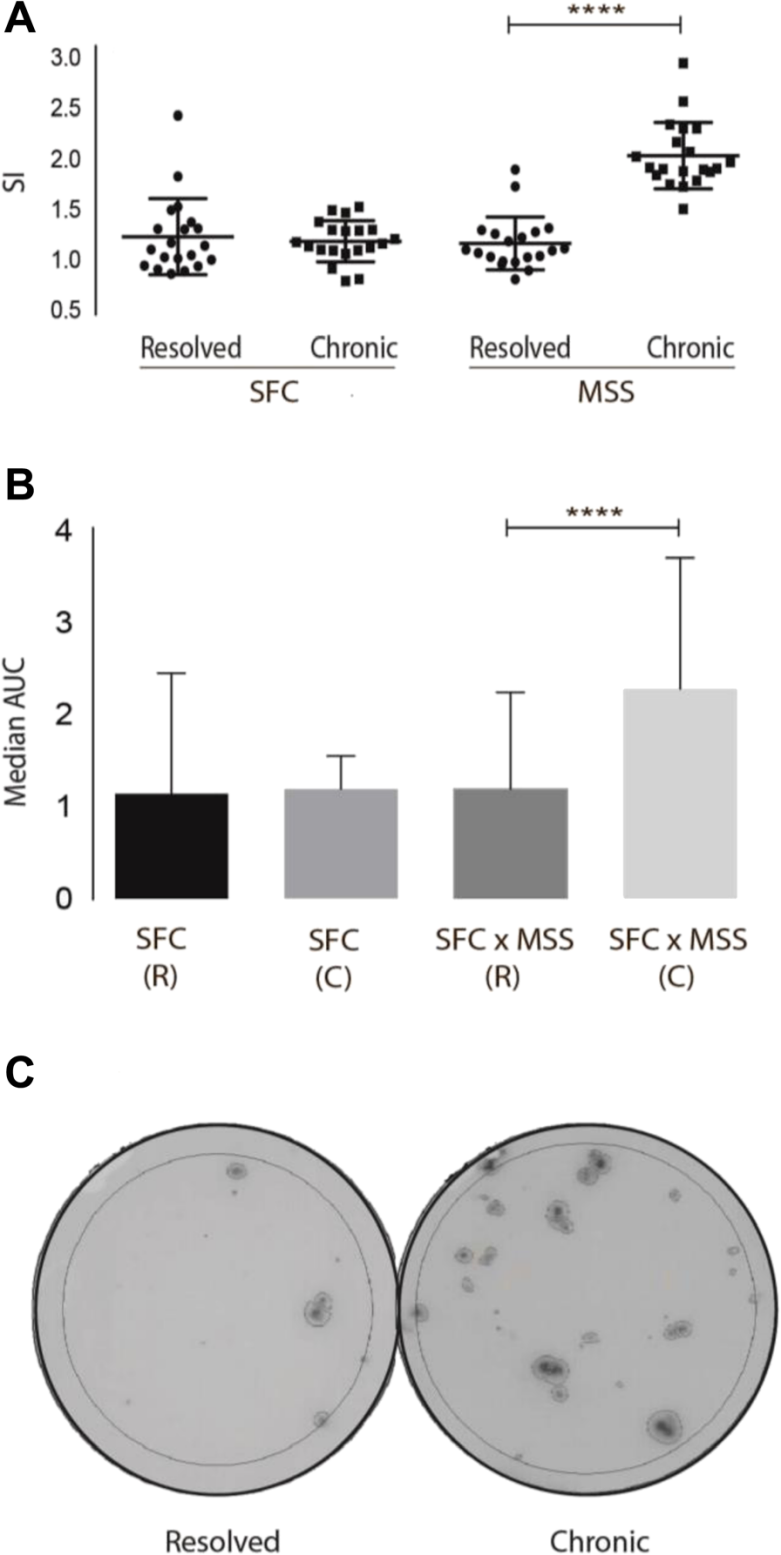
ELISPOT results of chronic and resolved HBV patients. **a** Stimulation index (SI) for CHB and RHB patients. Each datapoint represents the mean response to each individual epitope. No difference where found in the frequency of spot forming cells. The CHB patients had a significantly higher amount of IFN-γ production. Unpaired two-tailed *t*-test with equal SD, *N* = 10 RHB patients, *N* = 30 CHB patients, **** indicates *p* < 0.0001. **b** AUC showed that difference in response profiles did reach statistical significance when comparing SFC x MSS area, Dunn’s multiple comparisons test, R; resolved, C; chronic, **** Indicates *p* < 0.0001. **c** Picture from the data-analysis of a RHB and CHB patient. Large circle indicates the counted area. Small circles indicate the counted spots

**Fig. 2:**
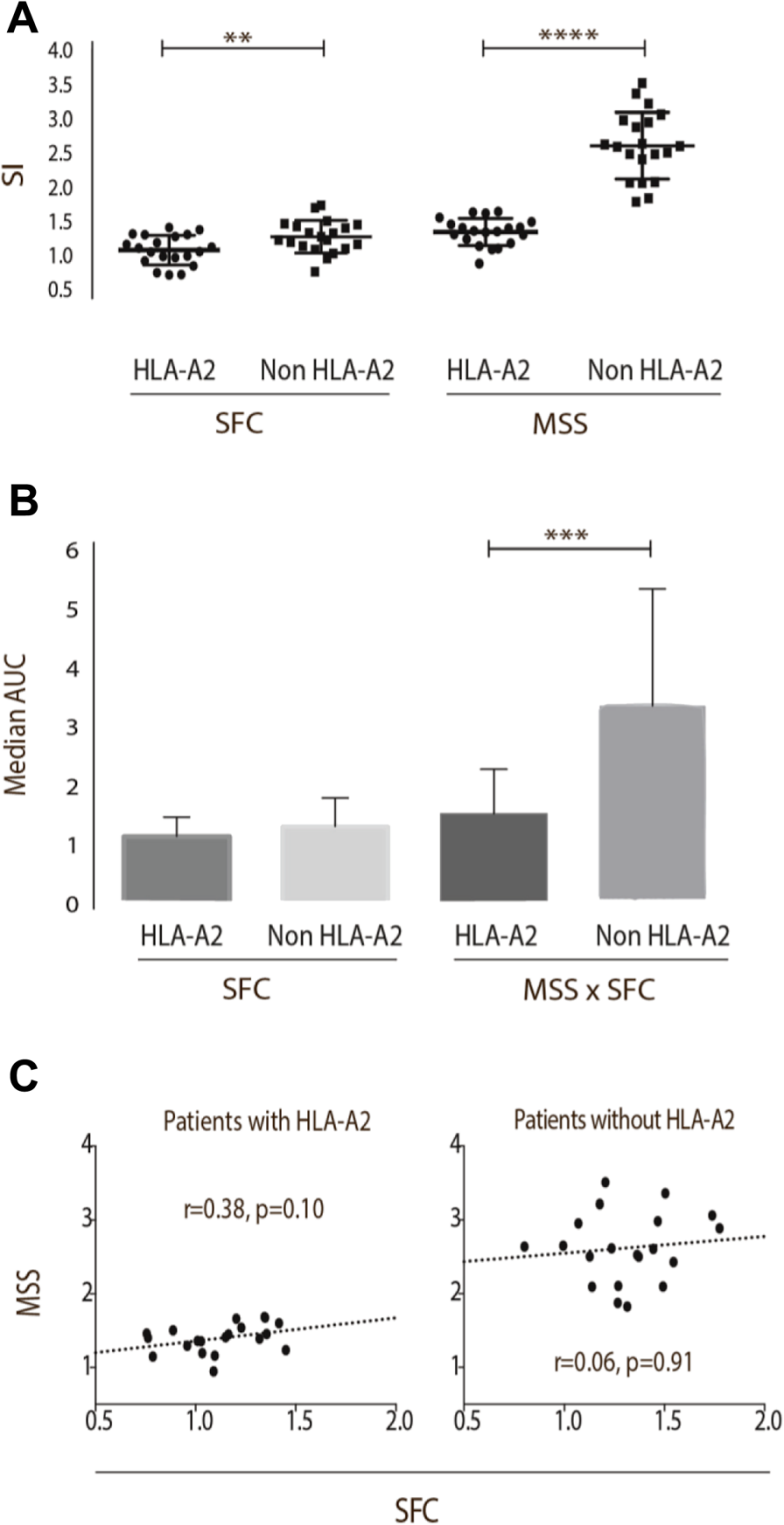
ELISPOT results of chronic HLA-A2 patients. **a** Stimulation index (SI) for CHB patients divided in two groups: Patients with the HLA-A2 genotype and patients without HLA-A2 genotype. Each datapoint represents the mean response to each individual epitope. The patients without HLA-A2 genotype had a significantly higher amount of IFN-γ production. If we included RHB patients in the analysis, the results were still significant. Unpaired two-tailed *t*-test with equal SD, *N* = 15 in each group, **** indicates *p* < 0.0001, ** indicates *p* = 0.0099. **b** AUC showed that difference in response profiles did reach statistical significance when comparing SFC x MSS area, Kruskal-Wallis (Dunn’s) multiple comparisons test, *** indicates *p* = 0.0009. **c** Spot number correlated with spot size. No significant correlation was found

**Fig. 3:**
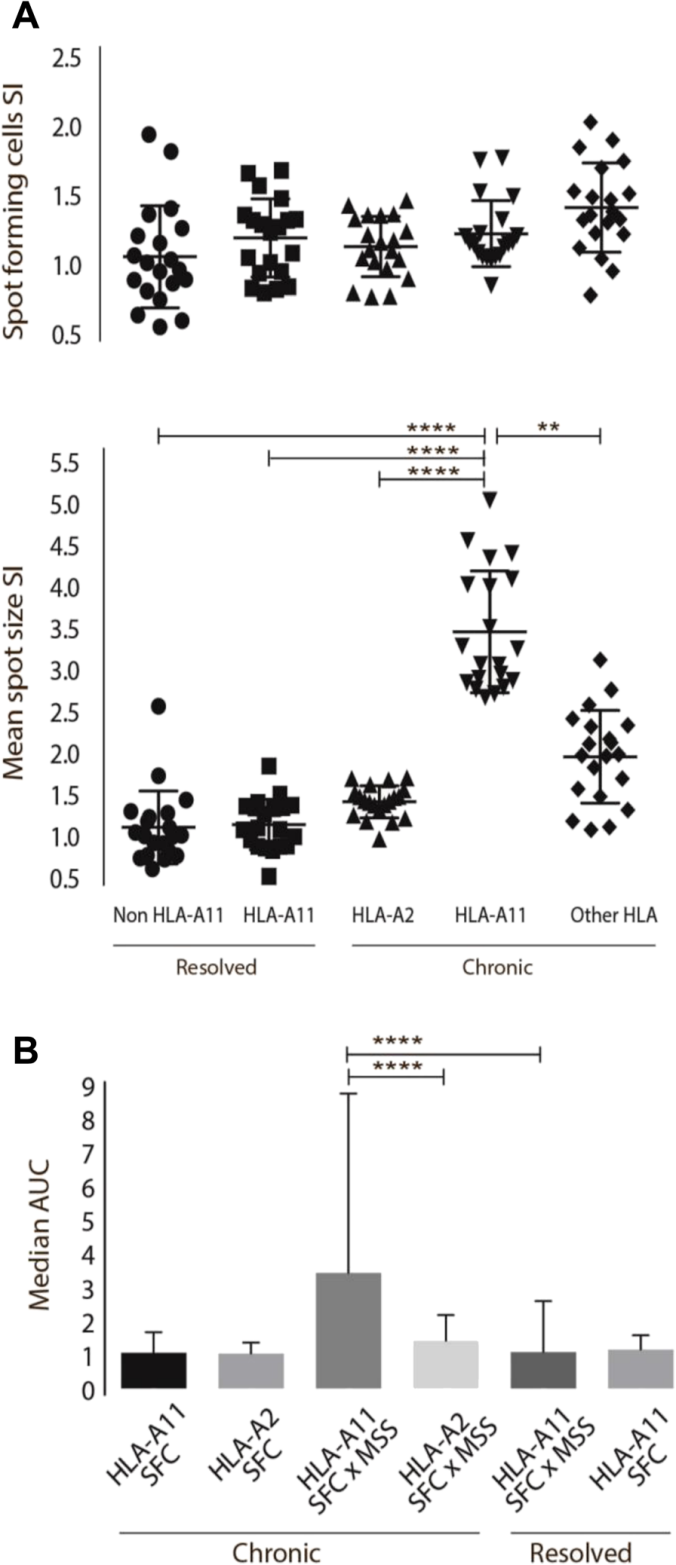
ELISPOT results of HLA-A11 and non-HLA-A11 patients. **a** Stimulation index (SI) for patients divided in 5 groups. Each datapoint represents the mean response to each individual epitope. CHB patients with HLA-A11 had significantly higher SI of MSS compared to all other group. No significance were found when analysis spot forming cells; R: resolved; C: chronic. Unpaired one-way ANOVA, Kruskal-Wallis (Dunn’s) multiple comparison test, Non HLA-A11 (R) (*N* = 8), HLA-A11 (R) (*N* = 2), HLA-A2 (C) (*N* = 15), Other HLA (C) (*N* = 10), **** indicates *p* < 0.0001, top line: 95 % CI of difference (−2.8;-1.9), middle line: 95 % CI (−2.7;-1.8), bottom line: 95 % CI (−2.4;-1.6), **: 95 % CI (1.1;1.9). **b** AUC showed that difference in response profiles did reach statistical significance when comparing SFC x MSS area, Kruskal-Wallis (Dunn’s) multiple comparisons test, **** indicates *p* < 0.0001

**Fig. 4:**
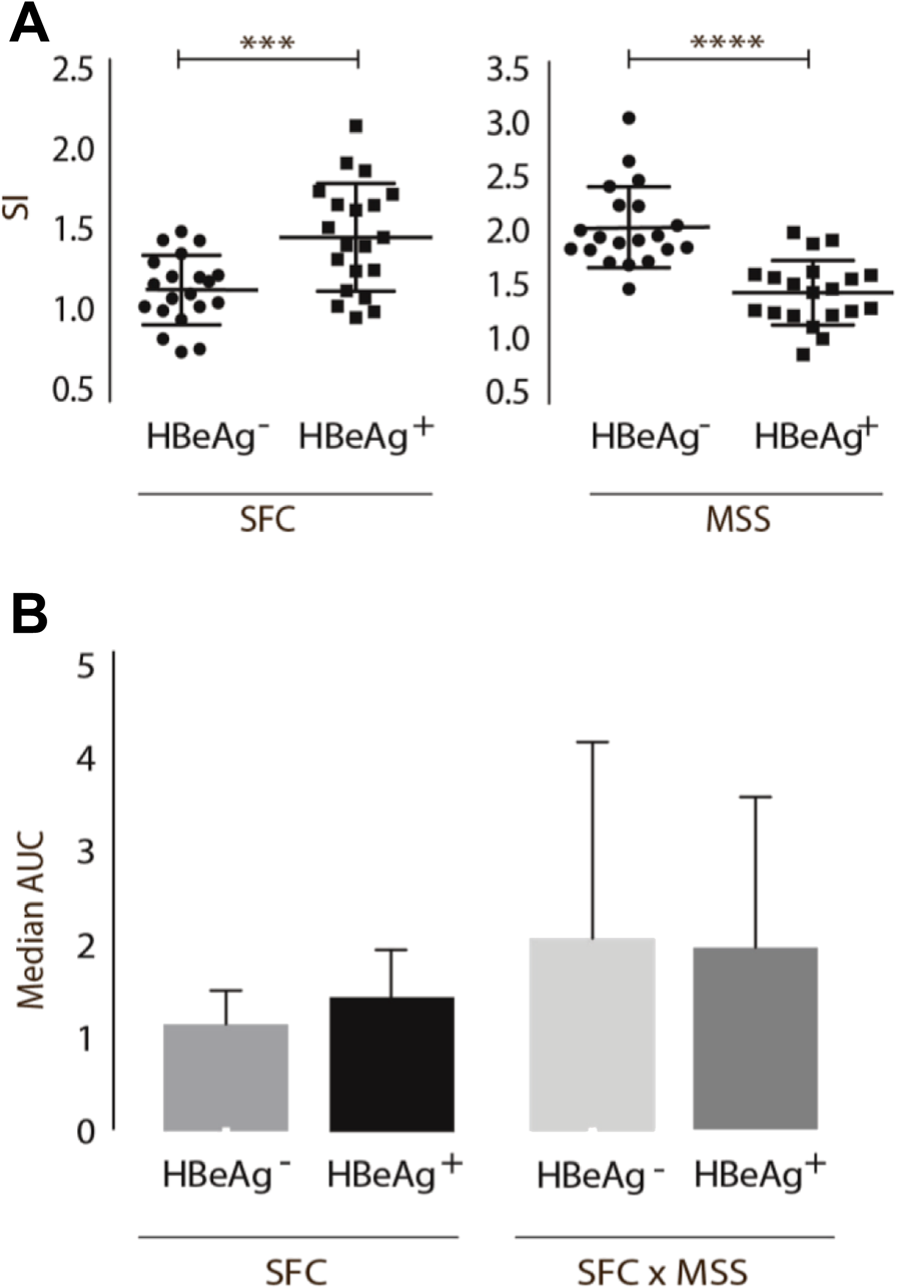
ELISPOT results of HBeAg positive and negative patients. **a** Stimulation index (SI) for CHB patients divided in HBeAg positive and HBeAg negative. Each datapoint represents the mean response to each individual epitope. CHB patients with HBeAg negative had significantly higher SI of MSS compared to HBeAg positive patients, and the contrary were found in number of spot forming cells, unpaired two-tailed *t* test with equal SD, HBeAg negative (*N* = 24), HBeAg positive (*N* = 6), **** indicates *p* < 0.0001, *** indicates *p* = 0.0009. **b** AUC did not reach statistical significant difference in Kruskal-Wallis (Dunn’s) multiple comparisons test

The numbers of HBV-specific T cells producing IFN-γ after stimulation with HBV epitopes were similar in CHB and RHB patients. Furthermore, the chronic patients surprisingly had a significantly higher MSS SI (Fig. 1). Comparison of the area under the curve (AUC) based on the response profiles of SFC and SFC x MSS, found that difference in response profiles did reach statistical significance when comparing SFC x MSS area (*p* < 0.0001). When stratifying the analysis for patients with HLA-A2 and non HLA-A2 genotype, the group of non HLA-A2 were found with significantly higher SI of SFC and MSS, the results being even more distinct when only the CHB patients were included in the analysis, MSS: *p* < 0.0001, SFC: *p* = 0.0099 (Fig. 2). AUC analysis confirmed the response profile to be significantly different. Analysing the SI stratifying for various HLA genotypes, only patients with HLA-A11 had significantly stronger SI of MSS (Fig. 3). AUC analysis of CHB patients with HLA-A11 genotype compared to RHB patients with HLA-11 genotype and CHB patients with HLA-A2 genotype respectively, revealed statistical significance in SFC x MSS response profile (*p* < 0.0001). Additionally, HBeAg positive patients, all with higher viral load, had a significantly lower SI of MSS, and none of the HBeAg positive patients had HLA-A11 genotype (Fig. 4).

### Correlations of SFC and MSS

To determine whether number of spots correlated with spot size, e.g. is the frequency of HBV specific T cells proportional with the functionality, we correlated SFC and MSS in all different groups. Using spearman’s correlation, no significance was found, but a trend towards correlation was found in all groups (data shown for chronic patients with/without HLA-A2 genotype in Fig. 2c).

### Computer prediction of immunoreactive epitopes

We compared the epitopes from the literature (experimentally confirmed, EC, epitopes 1–5 and 11–15) with epitopes computer predicted (CP, epitopes 6–10 and 16–20) to assess the ability of the computer algorithm to determine HBV epitopes. We compared the SI in 4 groups: Chronic and resolved HBV, HLA-A2 genotype and non HLA-A2 genotype, HLA-A11, HLA-A2 and other HLA genotypes, HBeAg positive and HBeAg negative patients. We analysed HBsAg and HBcAg epitopes together and separately, respectively, and found no significant difference in response towards epitopes experimentally confirmed compared with epitopes computer predicted.

Analysis of protein parameters included isoelectric point, length, instability index and aliphatic index and were performed for each epitope. We analysed the parameters in 4 groups: HBsAg epitope 1–5 EC, HBsAg epitope 6–10 CP, HBcAg epitope 11–15 EC, and HBcAg epitope 16–20 CP. No correlation between the different parameters and SI were found (data not shown). No significant difference where found between the groups (data not shown).

### Epitope assessment

We assessed the individual epitopes capability of eliciting specific T cell response, by the amount of IFN-γ produced. MSS SI varied widely within the groups. We found that 6 epitopes generally elicited a higher SI in all groups (Table 4). Three epitopes were already experimentally confirmed (EC-1, −4 and −11), and three epitopes where computer predicted (CP-7, −9 and −17).

**Table 4:** Epitope assessment

	HBsEC-1	HBsEC-4	HBsCP-7	HBsCP-9	HBcEC-11	HBcCP-17
Chronic	2,2	2,4	2,2	2,2	2,8	2
Resolved	1,1	1,2	1,8	1,6	1	0,9
HLA-A2	1,5	1,4	1,6	1,7	1,3	1,3
Non A2	2,9	3,1	2,6	2,7	3,1	2,6
Chronic HLA-A11	4,5	5	4,3	4,4	4	4
HBeAg positive	2	1,6	1,3	1,2	1,9	3
HBeAg negative	2,2	2,6	2,4	2,5	3	2,1

The assessment is based on stimulation index of mean spot size. HBsEC: HBsAg experimentally confirmed; HBsCP: HBsAg computer predicted; HBcEC: HBcAg experimentally confirmed; HBcCP: HBcAg computer predicted

## Discussion

Based on findings in several past studies, we hypothesized that a stronger IFN-γ response would be produced in PBMC from resolved HBV patients compared to PBMC from chronic HBV patients when stimulating with 20 individual HBV epitopes and quantifying IFN-γ response by of number of spot forming units and the amount of IFN-γ produced. Surprisingly, T cells from CHB patients recognized a similar number of CTL epitopes compared to T cells from RHB patients. We observed a relatively unimpaired cytokine response with higher levels of IFN-γ in CHB patients, in patients without HLA-A2 genotype**,** patients with HLA-A11 genotype, and we found lower levels of IFN-γ production in patients with high viral load (HBeAg positive). These results suggest that the impaired immune response in CHB is not solely due to impaired production of IFN-γ. Indeed, IFN-γ remains as an important component of the overall immune response required combating infection with HBV. Note that in our study the average stimulation index did not exceed 6, which still indicates a low IFN-γ production. Since the majority of epitopes were designed to give a strong response in patients with HLA-A2 genotype, with little difference among HLA-A*02 subtypes, and since we found a stronger response in patients with HLA-A11, this may suggest HLA-A11 is protective. No patients were both HLA-A11 and HBeAg positive and this supports HBeAg as a virulence factor and that control of HBV is more complex than T cell capability of IFN-γ production alone [26]. Other studies report that the amount of cytokine secreted by individual antigen specific T cells rather than differences in frequencies was the factor responsible for immune deficiencies in patients with HIV infection [27]. No studies have reported correlation between MSS and SFC in HBV infection. Most studies on HBV focus on SFC as the primary response parameter [28–30]. One tuberculosis vaccination study of non-human primates, found that spot size in combination with spot number, was key in determining a protective immune response [31]. We found no correlation between SFC and MSS respectively, but where able to describe similar results regarding SFC combined with MSS.

The three experimentally confirmed (EC) epitopes with highest SI (epitope 11 being HBcAg 18–27 which previously has been described as being highly immunogenic [10, 32, 33]) correlated with observations from previous studies. The three new computer predicted (CP) epitopes HBsCP-7, HBsCP-9 with predicted *K*_D_ < 50 and HBcCP-17 with predicted *K*_D_ > 500, may be worth examining in future studies. Surprisingly we saw a high SI when stimulation with HBsCP-17 despite the high *K*_D_ value, and this suggest that the CTL-inducing effect of an antigenic peptide not always correlate with the computer estimated *K*_D_ value [8, 9]. However, our results indicate that software both predict new epitopes as well as confirm previously described epitopes.

The liver cellular environment offers opportunities for immune evasion, which may partly explain why the immune response towards HBV frequently fails in the liver. Does this indicate HBV as a well-adapted pathogen - or is it the Th2 biased environment in the liver that permits the path to chronicity? The liver is an important site for T cell activation, however, the environment is biased towards induction of tolerance. This is partially due to the ongoing synthesis of IL-10 by cells constitutively exposed to traces of endotoxin and other microbial products [34]. In addition, the structure of the liver enables open access of naïve T cells to diverse subsets of antigen presenting cells (APCs). As a result, formation of memory T cell is lacking in this tissue since CD8^+^ T cell priming occurs without concomitant CD4^+^ T cell activation [35]. Furthermore, in the liver most APCs express PD-L1 providing the capacity to inactivate T cells. These mechanisms may be a key factor in the immunotolerance in chronic HBV in agreement with ours and the results of the investigations by others, this suggest that IFN-γ production is not the single major component in HBV clearance. Previous studies found a long lasting T cell response only after an acute infection [5, 6]. Our findings suggest, however, that these observations should be interpreted with caution. Memory CD8^+^ T cells can be divided into two subsets, namely central (T_CM_) and effector (T_EM_), which predominantly are found in the lymph node and in circulation, respectively. Nevertheless, their individual ability to confer protective immunity is not all clarified. One study found that T_CM_ were more efficient in mediating protective immunity resulting in antigen clearance, and that T_EM_ converts to T_CM_ in a lineage differentiation pathway. Furthermore, these results demonstrate that long-term persistence of memory T cells is primarily in the form of T_CM_ and that 400 days post infection, 95 % of all T cells were CCR7^hi^ [36]. This may explain our low response among the resolved HBV patients, since the blood was taken from the periphery, and all donors had been infected more than 2 years ago. Recently, Loggi et al. found the total breadth and magnitude of HBV specific T cell responses in IFN-γ ELISPOT did not differ significantly between groups of CHB and RHB patients [29] in accordance with our results.

We found evidence that HBeAg status and HLA-type affected the amount of IFN-γ production. IL-7 signaling is essential to CD8^+^T cell proliferation and function [37] and persistent viral antigen load suppresses CD127 expression, i.e., the α-chain on the IL-7 receptor on primed T cells. Studies have found that CD127 negatively correlates with serum HBV DNA and HBeAg levels in chronic HBV [38]. In addition to the impaired CD127 expression, exhausted HBV-specific T cells in the liver display increased PD-1 expression [39]. In several studies the PD-1/PD-L1 pathway has been shown to contribute to the suppression of HBV-specific T cell function and IFN-γ secretion is rapidly suppressed despite a continued presence of antigens. This loss of function coincides with the up-regulation of PD-1 [40]. We now report that a higher viral load in HBeAg positive patients influenced the number of HBV-specific T cells. Despite the higher number of SFC, however, the amount of IFN-γ produced was impaired compared to patients negative for HBeAg. This suggests that the IFN-γ producing subset of T cells may possibly be inactivated or exhausted by PD-1 linkage and CD127 down regulation while T cell ability to register and bind to the antigen is partly maintained.

To date, no HLA class I allele has been confirmed to be a protective or risk factor of CHB. However, many proposals have been made [41, 42] and, indeed, our findings are in support of some of these previous studies. The IFN-γ production was significantly higher in CHB patients with HLA-A11 genotype compared to the other groups, and with the highest SI levels observed among all groups tested (Fig. 3a). It has been reported previously that HLA-A11 may contribute in the development of natural immunity against HBV [43] and HLA-A11 were among the most common genotypes of responders to an HBsAg vaccine [44].

## Conclusion

In conclusion, our study suggests IFN-γ mean spot size as an important supplementary marker for T cell specificity, providing a measurement of the amount of IFN-γ when T cells fails to elicit response as calculated from number of specific T cells. This readout was found to enhance the discriminatory power of the ELISPOT assay and provide more information on the IFN-γ response profile of CHB and RHB patients. Furthermore, we showed that the computer program NetMHC version 3.2 8mer predictions, using Artificial Neural Networks Approximation could be a valuable tool in future studies regarding HLA genotypes and HBV epitopes. Hence, we found 3 immunogenic epitopes not previous described. Finally, our study highlights the role of the HLA-A11 allele in HBV infections with potential implication for both acquiring the disease and as inducing protective immunity by vaccination.

## Abbreviations

HBV: Hepatitis B Virus infection
IL-10: Interleukin 10
PD-L1: Programmed cell Death Ligand 1
CTL: Cytotoxic T lymphocytes
*K*_D_: Dissociation constant
MHC: Major Histocompability Complex
IFN-γ: Interferon gamma
ELISPOT: Enzyme-linked immunosorbent spot
CHB: Chronic Hepatitis B Virus infection
RHB: Resolved Hepatitis B Virus infection
HBsAg: Hepatitis B virus s-antigen
HBcAg: Hepatitis B virus c-antigen
HIV: Human ImmunodeficiencyVirus
HCV: Hepatitis C Virus infection
HBeAg: Hepatitis B virus e-antigen
anti-HBe: Antibodies against HBeAg
anti-HBc: Antibodies against HBcAg
anti-HBs: Antibodies against HBsAg
ELISA: Enzyme-linked immunosorbent assay
HBV DNA: Hepatitis B Virus deoxyribonucleic acid
PBMC: Peripheral Blood Mononuclear Blood Cells
PBS: Phosphate Buffered Saline
FCS: Fetal Calf Serum
DMSO: Dimethyl Sulfoxid
PCR: Polymerase Chain Reaction
AEC: 3-amino-9-ethyl carbazole
SFC: Spot Forming Cell (IFN-y producing cell)
MSS: Mean Spot Size
SI: Stimulation Index
ANOVA: Analysis of Variance
EC: Experimentally Confirmed
CP: Computer Predictions
AUC: Area Under the Curve
APC: Antigen Presenting Cells
T_CM_: Central Memory T cells
T_EM_: Effector Memory T cells
CCR7^hi^: C-C chemokine Receptor type 7
IL-7: Interleukin 7
CD127: alpha-chain of the IL-7 receptor
